# Risk-sharing agreements for medical devices: a stakeholder-based qualitative study in Czechia

**DOI:** 10.3389/fpubh.2025.1627299

**Published:** 2025-09-03

**Authors:** Petra Hospodková, Vladimír Rogalewicz

**Affiliations:** Department of Biomedical Technology, Faculty of Biomedical Engineering, Czech Technical University in Prague, Kladno, Czechia

**Keywords:** risk-sharing, medical devices, outcome based healthcare, post-transition healthcare systems, Central and Eastern Europe

## Abstract

**Objective:**

The rising costs and uncertain clinical value of innovative health technologies have spurred growing interest in alternative reimbursement models, particularly outcome-based risk-sharing agreements. While such models are increasingly discussed and applied in the pharmaceutical sector, their use in the context of medical devices remains limited and underexplored, despite similar challenges regarding evidence generation and financial risk. This qualitative study examines stakeholders’ perspectives and identifies key barriers to the implementation of outcome-based agreements for medical devices.

**Methods:**

A qualitative design was employed, based on 16 semi-structured interviews with stakeholders from payer organizations, providers, regulatory authorities, and the medical device industry. Interview transcripts were thematically analyzed based on the inductive coding principle.

**Results:**

Participants most frequently reported three dominant barrier domains: regulatory and legal uncertainty, weak infrastructure for real-world outcome measurement, and institutional reluctance to engage in shared-risk models. Nevertheless, respondents acknowledged the conceptual benefits of outcome-based agreements and supported the idea of small-scale pilot programs in selected high-cost therapeutic areas.

**Conclusion:**

Enabling broader adoption of such models requires not only regulatory and institutional evolution but also targeted investment in data infrastructure. In particular, the capacity to generate and interpret real-world evidence through Big Data analytics will be crucial for supporting sustainable, transparent, and evidence-based reimbursement decisions in the medical device sector.

## 1 Introduction

Long-term healthcare financing poses a challenge to all national healthcare systems of wealthy countries worldwide in their efforts toward ensuring sustainable healthcare (1). In recent years, the remarkable progress in diagnostic and therapeutic methods has brought about significant health benefits, albeit often associated with substantial costs. There is an agreement that, at least conceptually, the mostly used fee-for-service reimbursement systems should be replaced by outcome-based payments, which can fundamentally reduce low- or no-value care and simultaneously enhance overall care quality. This necessity becomes even more pronounced in the current climate of fiscal austerity, where budget constraints demand a more efficient allocation of resources (2, 3).

Moreover, countries in Central and Eastern Europe (CEE) face even greater fiscal pressures due to their lower GDP levels. Medical devices (MDs), typically imported at global market prices, represent a disproportionately high share of healthcare spending compared to historical EU countries, where healthcare expenditures are predominantly driven by personnel costs (4–6).

Medical devices naturally emerge as prime candidates for outcome-based reimbursement, for instance, through the application of risk-sharing agreements (RSAs). In most developed countries, before a MD is reimbursed, it is evaluated for its clinical effectiveness and cost effectiveness. Nevertheless, evaluating MDs poses considerably greater challenges compared to pharmaceuticals (7), which have a longer tradition of systematic assessment and are subjected to more rigorous and standardized evaluation methods in the pharmaceutical industry (8–12). Health technology assessment guidelines and methods are traditionally designed within the context of pharmaceutical reimbursement (13). Ciani et al. (14) noted that “existing regulatory processes for MDs generate less clinical evidence than the corresponding processes for drugs.” This evidence gap is further intensified by the specific characteristics of MDs: they have short life cycles, frequently undergo product modifications that may affect their efficacy and costs, often have multiple applications, and their efficacy may not only depend on characteristics of the MD itself but also on the skills and experience of those applying it in clinical practice (15). These factors collectively complicate clinical and economic evaluations, making their results not easily interpretable (16).

Healthcare payers (reimbursement agencies) must routinely grapple with high levels of risk and uncertainty. Thus, they hesitate to reimburse innovative MDs, which often leads to delayed access for patients to modern diagnostic and therapeutic methods, ultimately resulting in worsened health outcomes. The reasons are, above all, high cost of innovative technology and the lack of trust between manufacturers/distributors and healthcare payers regarding the efficacy and cost-effectiveness of these technologies. This dynamic becomes even more pronounced in CEE countries, which are characterized by lower GDP and lower wage and price levels. Beyond the economic pressures, institutional and operational challenges further complicate the ability of CEE countries to implement innovative reimbursement models. Although theoretical frameworks for outcome-based payments and RSA schemes have been well described internationally, their practical implementation remains limited in CEE, including the Czech Republic. Budgetary constraints, fragmented real-world evidence infrastructures, and specific administrative barriers exacerbate these challenges, making the Czech Republic a representative case study of a country with late technology adoption.

Risk-sharing agreements, also referred to as patient access schemes or managed entry agreements, represent arrangements between a payer (e.g., a hospital or insurance company) and a MD manufacturer, whereby the level or continuation of reimbursement is linked to the clinical or economic outcomes observed in a defined patient population over a specified period (17). RSAs have the potential to redistribute risk between manufacturers and payers, achieve cost savings, and simultaneously facilitate faster access to innovative technologies (18, 19).

These agreements offer an alternative to the conventional model based on the upfront payment of the full technology price (20). This study builds directly on the work of Drummond et al. (15) and Federici et al. (21), who critically examined the misalignment between the theoretical ideals and real-world implementation of RSA schemes. By focusing on the Czech Republic, this research provides an empirical contribution from a country where such models have remained largely theoretical and underutilized.

RSAs for MDs may differ significantly from those applied to pharmaceuticals. Key differences include the composition of payers (with greater involvement of hospitals and integrated healthcare systems), the nature of success or failure metrics (allowing for the use of short-term, process-related indicators), lower overall budgetary impacts, and a less developed evidence base at the time of market entry (22).

Several typologically distinct forms of RSAs exist, including Coverage with Evidence Development (CED), where reimbursement is conditional upon the collection of additional data, and Performance-Linked Reimbursement (PLR), where reimbursement is directly tied to the achievement of pre-specified clinical outcomes (22). Outcome-based contracts, performance-based contracts, and RSAs generally link reimbursement to the actual attainment of clinical benefits, thereby incentivizing higher quality of care and improved resource efficiency (20). Particularly in healthcare systems with constrained resources, such as those in CEE countries, innovative models like outcome-based contracts, delayed payment schemes, and RSAs can offer vital tools for balancing patient access to innovation with financial sustainability.

Matinheikki et al. (19) presents several examples of best practices—for instance, Medtronic offered a RSA for the antibacterial TYRX envelope used in pacemaker implantation, under which the company reimbursed hospitals for costs incurred in the event of post-operative infections. This strategy contributed to a broader adoption of the technology, which had demonstrated clinical benefits. Similarly, Biosense Webster (a division of Johnson & Johnson) introduced a risk-sharing program in the United States for the Thermocool radiofrequency ablation catheter, guaranteeing a discount if a repeat ablation procedure was necessary within 1 year. Likewise, Abbott implemented an agreement for its Quadra device for cardiac resynchronization therapy, offering a discount in the event of lead revision within the first year after implantation. Research findings further indicated that RSAs can increase the likelihood that physicians choose more innovative and expensive devices, while other decision-making groups (such as managers) tend to focus more on financial considerations.

Health economists have published numerous theoretical studies on the design and implementation of CED schemes. However, the practice of CED for MDs in Europe often deviates from the normative principles outlined in the health economics literature (15). Boriani et al. (20) points out that the implementation of RSAs carries both significant opportunities and substantial challenges. Opportunities include improved patient access to innovative treatments, short-term budget impact mitigation, outcome-based risk-sharing between stakeholders, generation of supplementary clinical evidence, and improvement of value-for-money considerations. Challenges involve administrative complexity, difficulties in monitoring patient outcomes, setting of measurable performance indicators, allocation of resources for data collection, and financial risks borne by manufacturers.

It can be concluded that professional societies, such as the European Heart Rhythm Association (EHRA), can play a pivotal role by supporting clinical evidence evaluation, promoting education around learning curves for innovative treatments, and advocating for the establishment of large registries to enable precise assessment of patient outcomes. While the number of RSAs for MDs is increasing in the United States (17), the adoption in Europe remains constrained by heterogeneous regulatory environments (6, 15). Nevertheless, the advent of Big Data and machine learning is expected to facilitate outcome monitoring and improve RSA feasibility, potentially leading to broader adoption and more refined decision-making frameworks. Overall, RSAs represent a growing trend in MD procurement strategies, offering the potential to enhance access to innovation and achieve a more favorable balance between clinical benefits and economic costs. However, their implementation remains complex and demands careful consideration of multiple dimensions and close collaboration among all involved stakeholders. Accordingly, this study aims to explore stakeholder perspectives on the feasibility and perceived barriers to implementing risk-sharing agreements and outcome-based payment models for MDs in the Czech Republic, thereby contributing to a better understanding of implementation dynamics in late-adopting healthcare systems.

## 2 Materials and methods

### 2.1 Qualitative research design

The qualitative component of this study was developed in accordance with established methodological standards for health services research, drawing in particular on the Consolidated Criteria for Reporting Qualitative Research (COREQ) framework (23). All semi-structured in-depth interviews were conducted by PH (woman—PhD in Public Health) and facilitated by external member of project team, both of whom have prior experience with qualitative research methods and are proficient in using MAXQDA for data coding. This approach ensures transparency and consistency in the planning, execution, and reporting of qualitative data. Neither interviewer had a prior personal or professional relationship with participants, and all respondents were informed of the researchers’ academic background and the study’s purpose before the interviews began, with reflexive journaling maintained to minimize bias. Semi-structured in-depth interviews were conducted with a purposive sample of stakeholders who possess direct experience with or influence over the use of risk-sharing agreements (RSAs) in healthcare. The study design aimed to capture rich, context-specific insights into the operational, strategic, and policy-related aspects of RSA implementation in the Czech Republic. This methodological strategy is consistent with best practices in qualitative health research (24–26), and aligns with previous empirical studies addressing RSA implementation (13, 15, 27, 28), allowing thus for meaningful contextualization and comparison.

### 2.2 Participant selection

A total of 16 stakeholders were selected using purposive sampling (29) to capture diverse, experience-based insights into the implementation of RSAs in the Czech healthcare system. Of 18 experts invited, 2 declined due to time constraints, resulting in 16 completed interviews. The sample was designed to ensure balanced representation from four key stakeholder groups: Healthcare Providers (HP), Insurance Representatives (IR), Policy Makers (PM), and Industry Experts (IE), with four participants in each category.

Selection criteria, detailed in Table 1, include professional role, minimum length of experience (10–20 years depending on stakeholder type), and direct engagement with RSAs or similar reimbursement mechanisms. Healthcare providers included both senior clinicians and hospital administrators involved in contract implementation; insurance representatives were executives responsible for coverage policy and strategic purchasing; policy makers included officials from the Ministry of Health, regulatory agencies, and HTA institutions; and industry experts, representing medical device and pharma companies, with experience in market access and managed entry agreements.

**Table 1:** Selection criteria for stakeholders.

Stakeholder group	Specific roles	Length of experience	Additional attributes
Healthcare providers (HP)	Doctors, hospital administrators	10+ years	Direct involvement in the implementation of RSAs
Insurance representatives (IR)	Executives, managers	15+ years	Experienced in negotiation and management of RSAs
Policy makers (PM)	Government officials, regulators, HTA specialists	20+ years	Influence on health policy and regulatory frameworks
Industry experts (IE)	Pharmaceutical company reps, medical device manufacturers	12+ years	Engaged in the development and marketing of products under RSAs

Participants were identified through professional networks, expert referrals, and public records of institutional affiliation and authorship on RSA-related topics. Recruitment followed a structured, multi-phase process: initial network referral, follow-up email invitation, and confirmation of eligibility based on inclusion criteria. All selected informants were actively involved in decision-making processes related to medical device reimbursement, health policy, or pricing negotiations. The purposive sampling approach ensured that all interviewees had relevant, first-hand experience with either the operational, regulatory, or strategic aspects of RSAs, thus increasing the credibility and contextual depth of the findings. This selection process followed a structured, multi-phase recruitment logic, aligning with best practices in qualitative health research as outlined by Bonisteel et al. (30).

### 2.3 Data collection method

The primary data collection method was semi-structured interviews providing a flexible yet comprehensive means of exploring the complex dynamics of RSAs. An interview guide was developed from an extensive literature review and pilot-testing with two external experts, leading to minor refinements in question wording and flow. Each interview was carefully structured to last approximately 60 min, accommodating diverse geographical locations of participants through the use of video conferencing tools. All interviews were audio-recorded with participant consent, and PH authored detailed field notes both during and immediately after each session. No repeat interviews were conducted. In all interviews, only the researcher and the participant were present; no third parties attended. This method not only facilitates a wide range of input, but also respects the time and contributions of each participant.

The development of the interview guide was informed by an extensive literature review, ensuring that all discussions were anchored in the relevant themes identified from existing research. Key areas covered in the interviews included:

- Stakeholders’ perceptions of the benefits and challenges associated with RSAs.
- Detailed accounts of experiences during contract negotiations.
- The observable impacts of RSAs on healthcare delivery.
- Constructive suggestions aimed at enhancing the effectiveness and efficiency of RSAs.

Data saturation was monitored and achieved after the 13th interview; the final three interviews served to confirm existing themes. Transcripts were returned to all participants for member checking, and 10 participants provided confirmation or minor clarifications.

Verbatim quotations are labeled R1–R16 and presented under each thematic sub-heading in the Results. Minor themes are supported by occasional direct quotes to ensure breadth of perspectives.

### 2.4 Data analysis

Data from the interviews were subjected to a rigorous process of thematic analysis, using MAXQDA 24 (Release 24.1.0) to facilitate the organization and coding of the transcript data. This method was chosen for its robustness in identifying, analyzing, and reporting patterns or themes within the qualitative data. The steps involved in the analysis were as follows:

1. *Thematic analysis:* The process began with a thorough reading of the interview transcripts, from which initial codes were generated directly from the data. This iterative coding process is sensitive to the nuances within the responses, allowing for the emergence of rich, detailed themes that accurately reflect the stakeholders’ experiences and opinions regarding RSAs.
2. *Coding procedure:* Two independent coders (PH and external member of project team) applied an inductive, *in vivo* coding approach; inter-coder discrepancies were discussed until consensus was reached, with an audit trail documenting all decisions. The resulting codes were then systematically clustered into broader thematic categories that reflected salient patterns across stakeholder responses.
3. *Verification*: A verification step involved a secondary review of the coding scheme and thematic structure by a third qualitative expert, ensuring reliability and validity of the findings.

This methodical approach to data collection and analysis ensured that the study not only captured a wide array of perspectives on RSAs but also has provided a deep, analytically sound understanding of how these agreements operate within the healthcare sector and their broader implications. As part of the visualization strategy, thematic maps were generated in which the size of each bubble reflects the frequency of code application. This graphical representation helps to illustrate the relative prominence of each thematic domain across stakeholder groups. This analytical strategy is consistent with established approaches to reflexive thematic analysis in health research (31, 32) and aligns with recent applications in qualitative studies of health policy and practice (33).

### 2.5 Ethical considerations

*Ethics committee consent*: The study was approved by the Ethics Committee of the Faculty of Biomedical Engineering, Czech Technical University in Prague under No. C47/2024 on 22 January 2024. The research was conducted in accordance with the local legislation and institutional requirements.

*Informed consent*: All participants were provided with an information sheet detailing the study’s purpose, the nature of their participation, and their rights, including confidentiality and the voluntary nature of their participation. Written consent was obtained from all participants.

*Confidentiality*: Measures were put in place to anonymize the identities of participants and the data obtained during the study to protect confidentiality and comply with relevant data protection laws.

## 3 Results

As a part of this qualitative research, key stakeholder perspectives on RSAs were systematically analyzed using open coding. The analysis identified four main thematic areas that emerged as central topics during the interviews: (I) Legislative Environment and Implementation Challenges, (II) Evaluation of Advantages and Disadvantages, (III) Barriers to Adoption, and (IV) Potential for Expansion in Healthcare. Stakeholders most frequently discussed the lack of clarity and specificity in the legislative framework, the critical importance of robust outcome measurement, and the complexity of financial risk management. Furthermore, much attention was given to organizational and technical barriers to adoption, particularly the inadequate data infrastructure and regulatory uncertainties. Respondents also emphasized the potential of RSAs to improve access to innovative treatments, enhance cost efficiency, and foster better patient outcomes. These findings offer a comprehensive insight into the factors shaping stakeholder attitudes toward RSAs and highlight the key areas that are to be addressed to enable their broader and more effective implementation.

### 3.1 Qualitative research

#### 3.1.1 Legislative environment and implementation challenges

The legislative framework governing RSAs is fundamental yet insufficiently robust to foster widespread adoption and effective implementation across the healthcare landscape, and particularly within medical device (MD) settings. This framework must address several critical aspects. **Clarity and Specificity:** Current legislation often lacks the specificity needed to guide the development and execution of RSAs effectively. Stakeholders express a need for clearer guidelines that delineate the responsibilities and expectations of all parties involved. For instance, there is an evident gap in defining measurable outcomes and the mechanisms for monitoring these outcomes, which are essential for the success of RSAs. **Regulatory Support:** The implementation of RSAs requires not only legislative backing, but also active support from regulatory bodies. This support should include the facilitation of negotiations, resolution of disputes, and oversight of the agreement terms to ensure compliance and fairness. As the interviewee R15 (Policy Maker) noted, *“the regulatory environment needs to evolve to provide a nurturing ground for RSAs, where innovation is encouraged, while maintaining strict adherence to patient safety and care quality standards.”* **Adaptability and Responsiveness:** Healthcare is a rapidly evolving field, and legislation related to RSAs must be adaptable to keep pace with medical advancements and market dynamics, especially considering the dynamic development of MDs. The legal framework should allow for modifications of agreements as new treatments become available and as more data on their effectiveness and cost-effectiveness is gathered.

**Figure 1:**
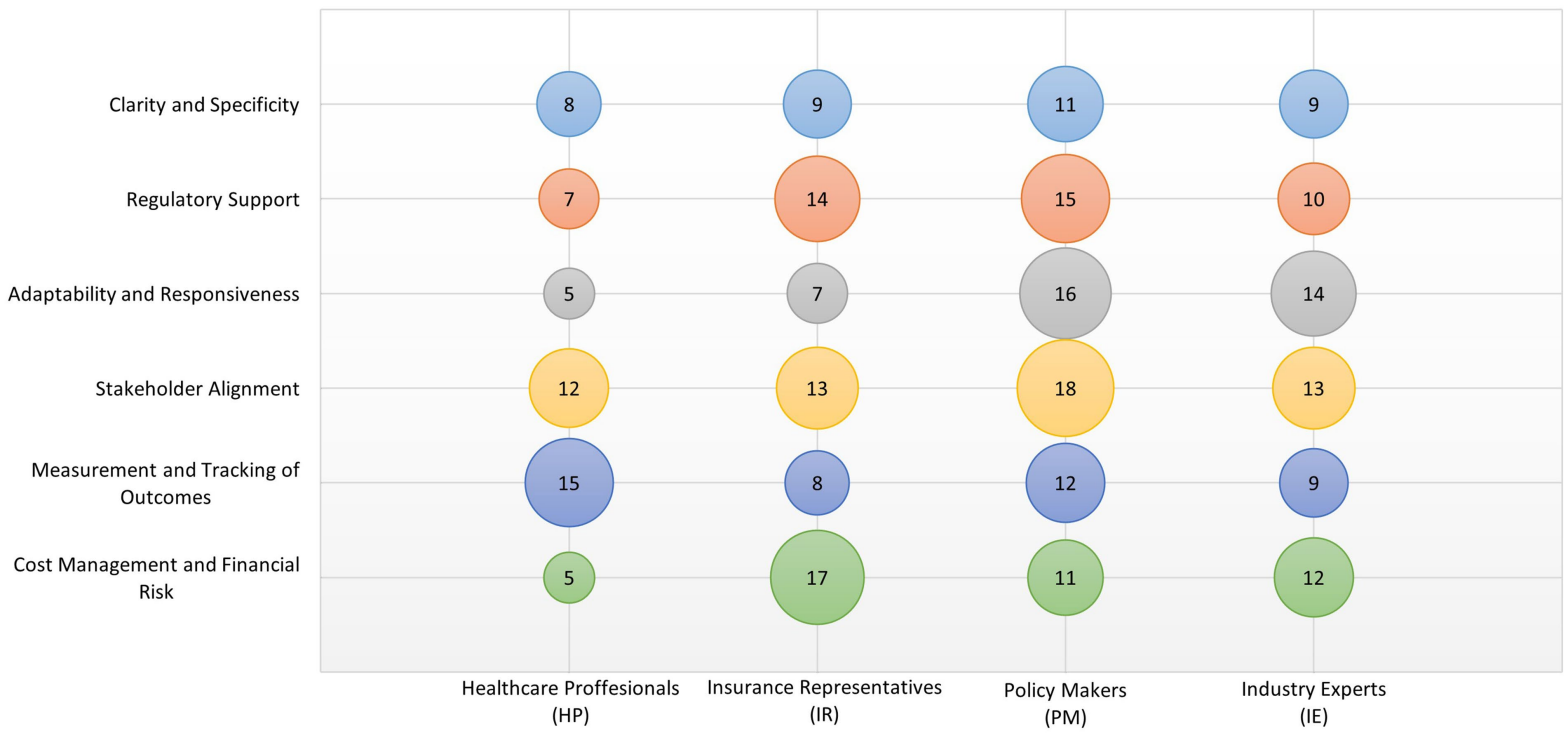
Distribution of stakeholder attention across six thematic categories related to legislative and implementation issues. Each row corresponds to a thematic category; each column represents one of the stakeholder groups: Healthcare Providers (HP), Insurance Representatives (IR), Policy Makers (PM), and Industry Experts (IE). The size of each bubble reflects the frequency of code occurrence. Equal representation was ensured for all groups (*n* = 4 per group).

Implementing RSAs involves multiple stakeholders, each with distinct interests and expectations. The complexity of these agreements poses several challenges. **Stakeholder Alignment:** One of the primary challenges in the implementation of RSAs is achieving alignment among diverse stakeholder groups, including healthcare providers, payers, and pharmaceutical companies, as well as manufacturers of MDs. Each group has different priorities and risk tolerances, which can complicate negotiations and agreement terms. Effective RSAs require a balanced approach that considers the financial, clinical, and operational impacts on all parties. **Measurement and Tracking of Outcomes:** A significant implementation challenge is the development of agreed-upon metrics for measuring treatment outcomes. These metrics must be scientifically valid, clinically relevant, and economically feasible to monitor. Furthermore, the integration of these metrics into existing healthcare data systems poses technical challenges. Stakeholder R2 (Healthcare Provider) highlighted the difficulty of “*establishing reliable and consistent methods for tracking patient outcomes across different healthcare settings, populations and MDs used in clinical practice.*” **Cost Management and Financial Risk**: RSAs are fundamentally designed to manage financial risk by linking payment to clinical outcomes. However, setting up the financial structures that support risk distribution between stakeholders can be complex. There is often a need for upfront investment from one or more parties before the benefits of the RSA are realized, which can deter participation. Additionally, managing the costs and associated risks requires sophisticated financial models and robust forecasting capabilities. Figure 1 presents the distribution of stakeholder focus on thematic categories related to legislative and implementation issues.

#### 3.1.2 Evaluation of advantages and disadvantages

The evaluation of the advantages and disadvantages of RSAs is essential to understand their potential impact on healthcare systems including those related to MDs. This detailed examination helps stakeholders weigh the benefits against the challenges, providing a balanced perspective necessary for informed decision-making. Here is an expanded analysis of the advantages associated with RSAs: **Improved Access to Innovative Treatments:** RSAs can facilitate faster access to new and potentially life-saving treatments that might otherwise be delayed due to cost concerns. By aligning the payment structure with clinical outcomes, these agreements can enable healthcare systems to adopt innovative therapies without bearing the full financial risk upfront. This is particularly advantageous for treatments that have high potential but come with significant uncertainty regarding long-term effectiveness. **Cost Management and Efficiency:** RSAs are designed to link the cost of treatments to their actual clinical outcomes, which can lead to more efficient use of healthcare resources. This model encourages healthcare providers and payers to focus on treatments that offer the best value for money. As the interviewee R3 (Healthcare Provider) highlighted, *“RSAs introduce a financial model that prioritizes patient outcomes over volume, driving overall cost efficiency in healthcare delivery.”* **Enhanced Patient Outcomes:** By incentivizing the achievement of specific health outcomes, RSAs encourage healthcare providers to optimize treatment protocols and follow-up care. This focus on outcomes rather than just service delivery helps improve the quality of care provided to patients, potentially leading to better health outcomes.

**Figure 2:**
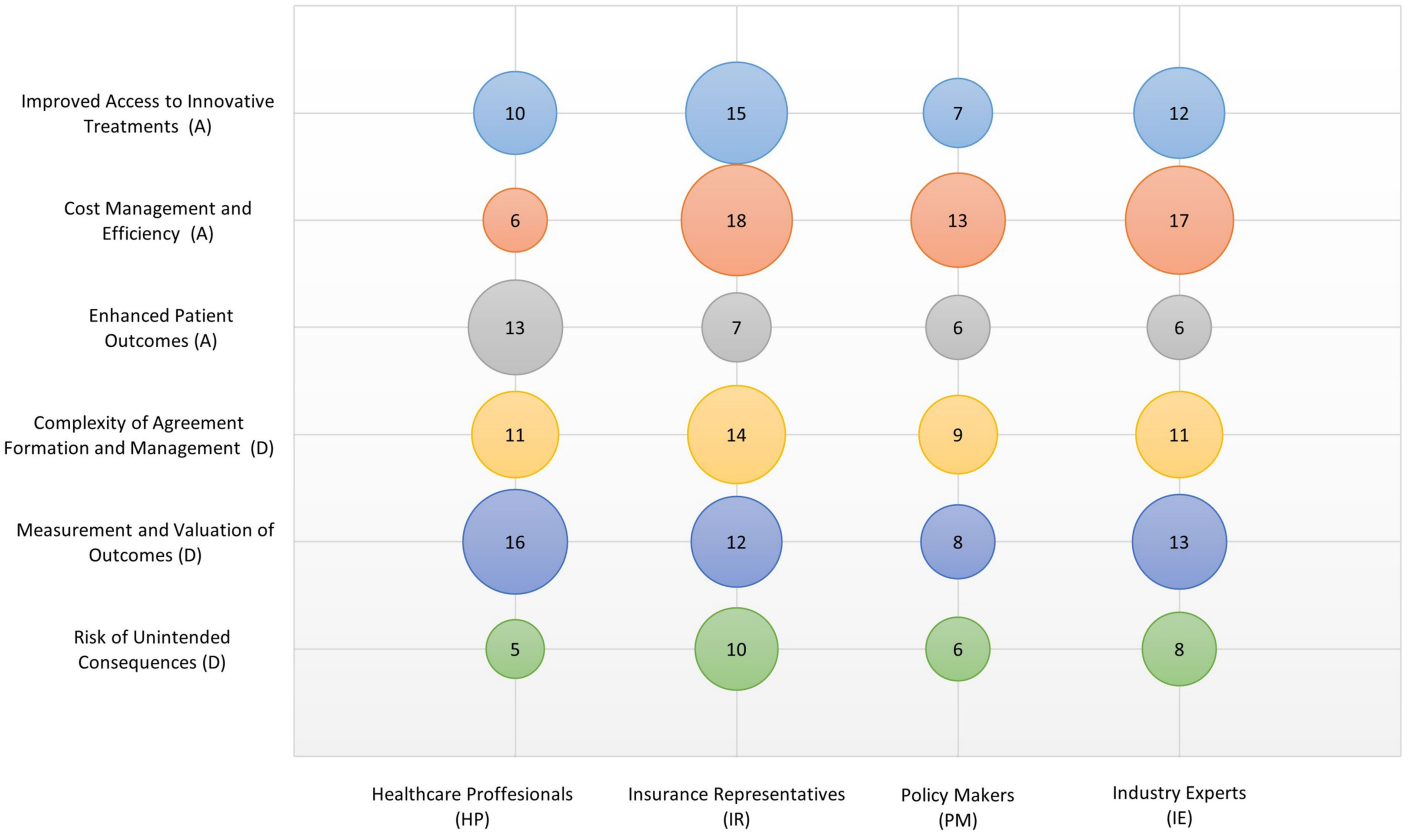
Stakeholder perspectives on selected benefits and drawbacks of risk-sharing agreements. Thematic categories labeled (A) indicate advantages; those labeled (D) refer to disadvantages. Bubble size represents the number of references assigned to each theme by the respective stakeholder group. Each group consisted of four participants (*n* = 4 per group).

The analysis of disadvantages of RSAs includes for example **Complexity of Agreement Formation and Management:** The negotiation and management of RSAs can be highly complex due to the need to define specific outcomes, measurement methods, and the conditions under which payments will be made. This complexity can lead to significant administrative burdens and may require substantial time and expertise to manage effectively. The challenge is articulated by the stakeholder R4 (Industry Expert) who noted that *“the administrative overhead involved in setting up and managing RSAs can be prohibitively high, detracting from their potential benefits.”* **Measurement and Valuation of Outcomes:** One of the critical challenges in implementing RSAs is the difficulty in measuring and valuing the outcomes upon which payments are based. Determining what constitutes a successful outcome and its accurate measuring can be fraught with technical and ethical challenges. There are also concerns about how to adjust for external factors that might influence outcomes, but are beyond the control of healthcare providers. **Risk of Unintended Consequences:** While RSAs aim to align incentives toward better health outcomes, there is a risk of unintended consequences if the agreements are not carefully designed. For example, if not appropriately structured, a RSA might incentivize treatments that are more profitable rather than those that are most beneficial to patients. Additionally, there is a potential for such agreements to discourage the treatment of patients who are less likely to achieve the defined outcomes due to underlying health conditions. Figure 2 illustrates stakeholder perspectives on selected benefits and drawbacks of risk-sharing agreements.

#### 3.1.3 Barriers to adoption

The adoption of RSAs in healthcare encounters several significant barriers including those specific to the field of MDs. Understanding these obstacles is crucial for stakeholders aiming to implement RSAs effectively. Here is a detailed analysis of the primary barriers to the adoption of RSAs, highlighting the complexities involved and potential solutions.

The first and most important barriers were called Financial and Organizational Barriers, which cover: **Upfront Costs and Financial Risk**: One of the primary groups of barriers to the adoption of RSAs are financial and organizational ones, including significant concerns about the financial risks associated with upfront costs. Healthcare providers and payers may hesitate to commit to agreements that require substantial initial investments without guaranteed outcomes. This is particularly challenging in environments where budget constraints are stringent, and financial stability is paramount. As noted by R5 (Healthcare Provider), *“the uncertainty of financial returns from RSAs makes it difficult for institutions to justify the initial expenditure, especially under strict budgetary constraints.”* **Institutional Resistance**: There is often resistance within institutions to adopting new payment models, especially those that involve complex monitoring and reporting requirements. Many healthcare organizations operate within traditional financial structures that favor established payment mechanisms. Changing these entrenched systems requires not only logistical adjustments but also a shift in organizational culture, which can be a slow and challenging process.

Technical and Data-Related Barriers were also named, for example: **Lack of Adequate Data Systems**: Another significant group of barriers are technical and data-related ones. Effective RSAs depend heavily on robust data systems to track outcomes and measure performance accurately. In many healthcare settings, the necessary infrastructure for collecting and analyzing this data is either underdeveloped or absent. The challenge is even more pronounced for MDs, where continuous performance monitoring and data integration are critical, but often insufficient. This gap makes it challenging to implement RSAs effectively, as stakeholders lack the tools to monitor compliance and measure success reliably. **Complexity of Outcome Measurement**: Defining and measuring outcomes in a healthcare context is inherently complex. Outcomes can vary widely depending on the patient population, secondary diagnoses, treatment conditions, and other external factors. Developing universally accepted and measurable outcomes for RSAs is a significant challenge that can impede their adoption. A Policy Maker (R6) emphasized that *“defining clear, measurable outcomes that are agreed upon by all parties is often a contentious and challenging process.”*

**Figure 3:**
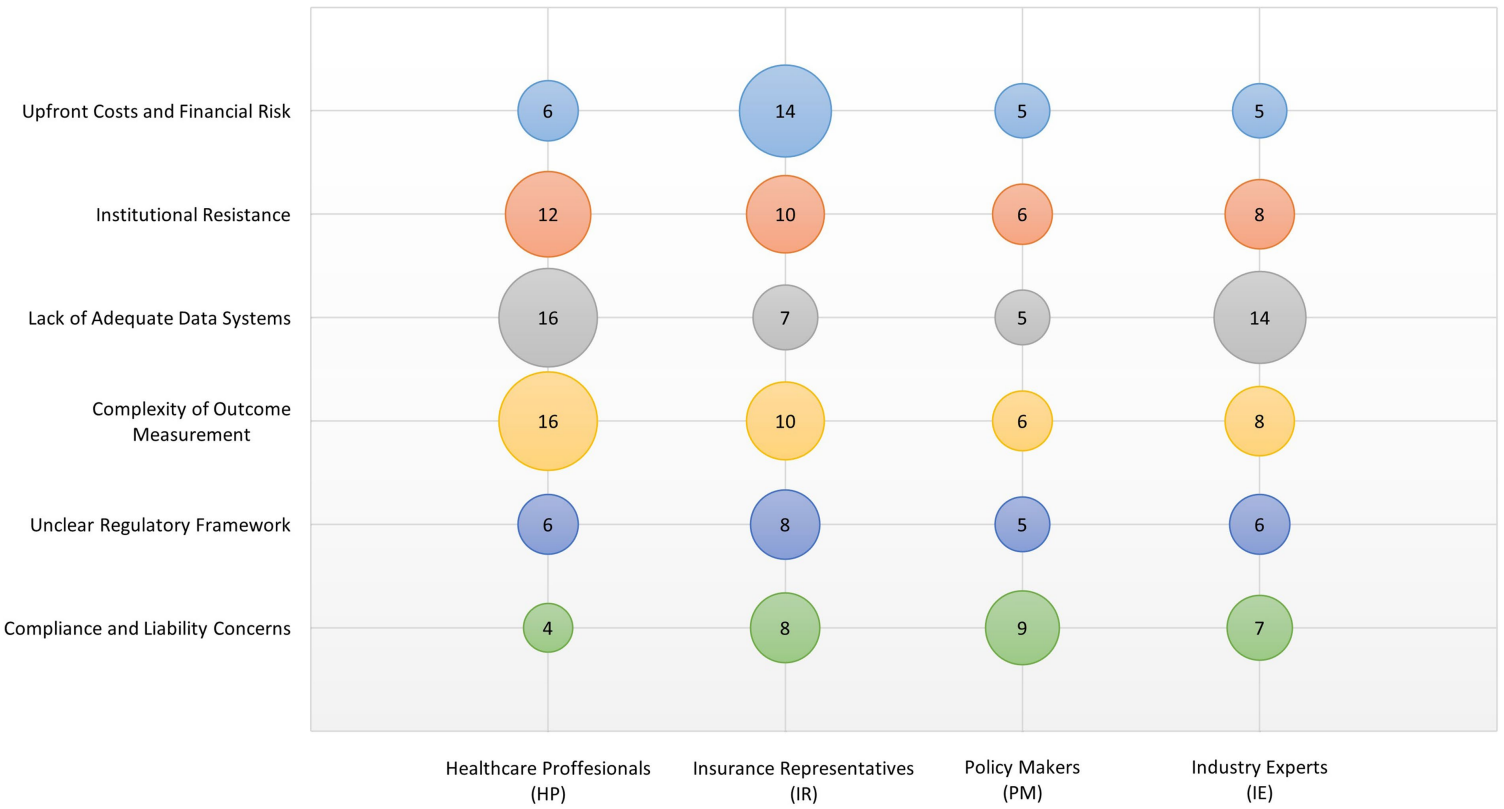
A visualization of how frequently each stakeholder group emphasized specific barriers to RSA implementation. The thematic categories span financial, institutional, technical, and legal concerns. Bubble size reflects the frequency of thematic references. All stakeholder groups were equally represented (*n* = 4 per group).

Finally, regulatory and legal barriers were named: **Unclear Regulatory Framework**: The legal and regulatory frameworks governing RSAs are often unclear or insufficiently developed. This lack of clarity can create legal uncertainties that deter stakeholders from entering into RSAs. Regulatory bodies may also lack the experience and guidelines on how to effectively supervise these agreements, which leads to a cautious approach among potential participants. **Compliance and Liability Concerns**: There are significant concerns about compliance with existing healthcare laws and regulations, including those related to patient privacy, data security, and anti-corruption statutes. Additionally, liability issues can arise if outcomes do not meet the expectations set in the RSAs, potentially leading to disputes and legal challenges. These concerns were articulated by R7 (Insurance Representative) who noted that “*navigating the compliance landscape while trying to implement innovative contractual agreements like RSAs is a major barrier, fraught with potential legal pitfalls*.” Figure 3 visualizes how frequently each stakeholder group emphasized specific barriers to RSA implementation.

#### 3.1.4 Potential for expansion in healthcare

The potential for expanding the use of RSAs in healthcare is significant and extends importantly to the domain of MDs. These agreements present an opportunity to address some of the systemic challenges within healthcare systems by aligning costs with outcomes. However, realizing this potential requires overcoming a number of various complexities and challenges associated with RSAs.

The potential for broader application in healthcare was very often presented: **Exploring the Expansive Reach of RSAs across Medical Fields**: One key area of potential for RSAs lies in their broader application across various medical fields and treatments. MDs, in particular, represent a promising area for expanding RSAs due to the need to verify real-world performance and clinical effectiveness before committing significant financial resources. These agreements can be particularly beneficial where treatments are highly innovative yet come with significant uncertainties regarding their long-term effectiveness and costs. RSAs can help mitigate these uncertainties by tying reimbursements to actual clinical outcomes, facilitating thus earlier access to cutting-edge therapies. As healthcare continues to advance rapidly, the flexibility of RSAs to adapt to new treatments and technologies becomes increasingly valuable. **Driving Cost Efficiency and Healthcare Sustainability**: RSAs have the potential to drive cost efficiency in healthcare by ensuring that payment mechanisms are directly linked to treatment outcomes. This can lead to more prudent spending in healthcare, as funds are allocated based on the effectiveness of interventions rather than the volume of services provided. This is equally relevant for MDs, where cost-effectiveness and real-world benefit are critical considerations. This shift not only has the potential to reduce wasteful expenditures but also to improve overall healthcare sustainability by promoting treatments that provide real value to patients.

In our research, enhancing patient outcomes through focused care was also mentioned: **Fostering Improved Health Outcomes**: Another significant potential of RSAs is their ability to enhance patient outcomes. By incentivizing healthcare providers to focus on achieving specific health outcomes, RSAs encourage the adoption of best practices and innovative care models that are tailored to patient needs. This focus on outcomes can lead to improvements in care quality, patient satisfaction, and overall health results. For MDs, outcome-based models can accelerate the integration of truly beneficial innovations into clinical practice, thereby enhancing patient care. Moreover, RSAs can support personalized medicine approaches by aligning financial incentives with customized patient care strategies, thereby potentially transforming patient management in various chronic and acute conditions.

**Figure 4:**
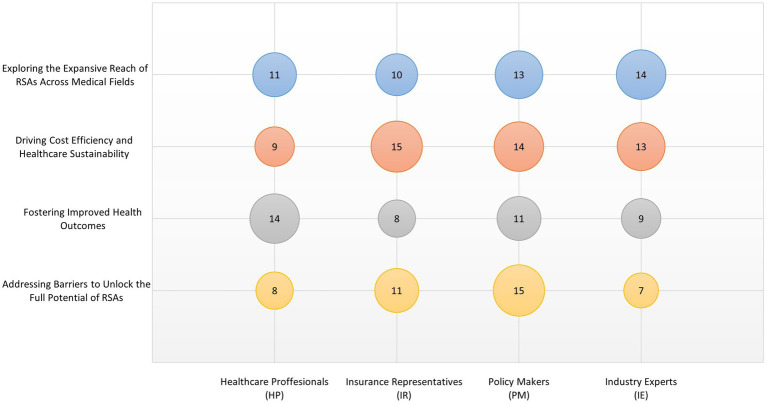
Stakeholder views on areas where RSAs could be further applied or expanded. Thematic categories include strategic and outcome-related dimensions. The size of each bubble indicates the relative frequency of coded references. Each group of stakeholders included four experts (*n* = 4 per group).

The respondents also described overcoming implementation challenges to realize the full potential of risk-sharing: **Addressing Barriers to Unlock the Full Potential of RSAs**: While the potential benefits of RSAs are clear, their widespread adoption is hampered by several implementation challenges, as discussed above. To fully realize the potential of RSAs in expanding healthcare, it is crucial to address these barriers effectively. This includes enhancing data capabilities, refining legislative frameworks, and fostering a cultural shift toward outcome-based models in healthcare institutions. Moreover, continuous education and engagement with all stakeholders—providers, payers, patients, and policymakers—are essential to overcome skepticism and build trust in RSAs. Figure 4 summarizes stakeholder views on areas where RSAs could be further applied or expanded.

## 4 Discussion

This study highlights a fundamental disconnect between the theoretical promise of RSAs and the operational context in the Czech Republic. The findings show that regulatory ambiguity, institutional fragmentation, and weak outcome measurement systems inhibit RSA deployment. These observations are consistent with the broader literature on the barriers to managed entry agreements (34, 35), particularly in less mature healthcare systems (36). The stakeholders’ concern about the absence of measurable outcomes echoes previous warnings that without robust data infrastructure, outcome-based models risk failure in implementation (37). Against this backdrop, emerging digital infrastructures and technologies—particularly the European Health Data Space (EHDS) and applications of Artificial Intelligence—offer a promising avenue to overcome current data limitations and enable robust outcome measurement. As highlighted by Pecchia et al. (38), data are becoming the new strategic resource in medicine, and AI is seen as a key enabler for unlocking their potential to support evidence-based policy-making and outcome-driven regulation in the medical device domain. This is particularly relevant in Central and Eastern Europe and the Middle East, where recent research by Ádám et al. (4, 5) has systematically documented barriers to implementing both outcome-based reimbursement and delayed payment models, including administrative burden, insufficient IT infrastructure, and fragmented governance frameworks.

Our findings are in line with Drummond et al. (15), who stress that European RSA practice often fails to reflect the theoretical models developed by health economists. The Czech case is an example of this discrepancy in its most extreme form, where theory remains entirely unimplemented. Similarly, Kovácz et al. (6) noted that in CEE countries, the lack of a supporting legislative and technical infrastructure inhibits RSA uptake. In contrast, Carlson et al. (17) and Chen et al. (22) document that in the U. S., the progress was driven by a combination of regulatory flexibility and the existence of clinical data systems capable of capturing short-term performance indicators.

For the Czech Republic, the results suggest that the foundation for RSAs in MDs must be established at the regulatory and data infrastructure levels. As Makady et al. (37) emphasize, the generation of real-world evidence for MDs is complicated by fragmented data sources, lack of interoperability, and weak registry coverage—issues that are all present in the Czech setting. Before RSA pilots can be launched, the Ministry of Health and HTA bodies should consider creating formal guidance on which outcomes are acceptable and how evidence should be collected.

This study highlights several barriers identified in international literature as well: rapid incremental innovation, difficulty of standardizing endpoints, operator-dependent performance, and lack of local evaluative capacity (15, 39). Czech stakeholders particularly emphasized the “cost of administration” and legal uncertainty—issues previously reported in both European and North American RSA experience with MDs (22).

Despite these constraints, Czech stakeholders did not reject RSAs categorically. Many agreed that RSAs may be feasible in narrow technology areas—particularly implantable devices in cardiology, neuromodulation, or oncology diagnostics. As Chen and Carlson (22) observed, early PBRSAs for MDs in the US were often led by integrated care systems with data capacity and focused on short-term outcomes. A similar approach could be piloted in the Czech Republic using conditional continuation contracts tied to registry participation or post-market surveillance data.

The Czech context would benefit from small-scale pilot projects that simulate RSA designs under real regulatory and institutional constraints. These pilots could draw on the decision tool proposed by Kovácz et al. (6) for late adopter countries, which includes guidance on when and how to transfer RSAs from Western European settings. Additionally, evaluation of cost-effectiveness uncertainty could benefit from simplified Value of Information (VOI) approaches, adapted, however, for settings with limited HTA and modeling capacity (15).

This study has several limitations. First, it is based on a purposive sample of 16 stakeholders and captures only qualitative perceptions. Although thematic saturation was achieved, the findings are not statistically generalizable. Second, the use of thematic maps and code frequency visualizations serves illustrative purposes only and does not imply inferential comparisons across stakeholder groups. These visual representations are intended to support qualitative interpretation, not to validate differences with statistical significance.

Nevertheless, this exploratory study offers a valuable foundation for follow-up research. Future studies should include legal experts, hospital administrators, and health data managers to further map the readiness of regulatory and technical systems for RSA implementation. Building on the themes identified here, subsequent research could adopt a mixed-method design integrating stakeholder surveys, legal feasibility assessments, and registry data audits. In particular, structured stakeholder questionnaires could test the prevalence and strength of attitudes observed in this study, and compare them across regions, professions, and types of institutions.

Although the Czech Republic does not yet possess the prerequisites to scale RSAs for medical devices, early steps can and should be taken. These include legal clarification, targeted investment into registries, and institutional learning through pilot initiatives. Lessons from early technology adopter countries (6) countries and selective transfer of tested frameworks could accelerate progress.

## 5 Conclusion

This study offers the first in-depth, stakeholder-based exploration of outcome-based risk-sharing agreements for medical devices in the Czech Republic. We identified three core barrier domains—regulatory and legal uncertainty, weak real-world evidence infrastructure, and institutional resistance—and highlighted the strategic potential of focused RSA pilots in targeted therapeutic areas. Our findings underscore the urgent need for clear legislative guidance, investment in robust data and registry systems, and collaborative frameworks to support sustainable, evidence-driven reimbursement practices in Central and Eastern European healthcare settings.

## Data Availability

The raw data supporting the conclusions of this article will be made available by the authors, without undue reservation.
